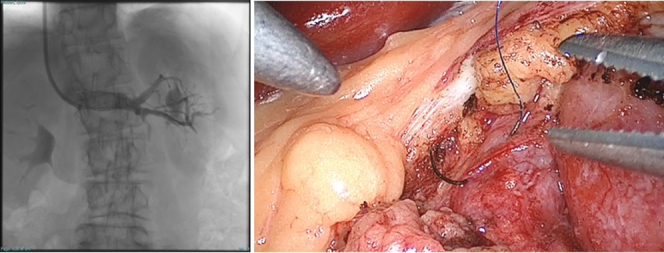# Hybrid approach for recurrence nutcracker syndrome involving left renal vein stenting with laparoscopic stent external fixation

**DOI:** 10.1016/j.jvscit.2025.101739

**Published:** 2025-01-15

**Authors:** Mattia Mirandola, Bruno Migliara, Gaetano Grosso, Rossella Bertoloni, Andrea Griso, Daniele Bissacco

**Affiliations:** a Department of Vascular Surgery, Ospedale Pederzoli, Peschiera del Garda, Italy; b Department of Urology, Ospedale Pederzoli, Peschiera del Garda, Italy; c Department of Clinical Sciences and Community Health, University of Milan, Milan, Italy

A 39-year-old women was admitted to our department experiencing chronic, recurrent pain in her left inguinal region. Notably, she has undergone a surgical procedure involving the transposition of the left renal vein to cava vein for Nutcracker syndrome treatment. Additionally, she has a history of May-Thurner syndrome, for which she underwent left iliac vein stenting, venous embolization for extensive endometriosis, as well as for a hemorrhagic corpus luteum cyst, and has had two laparoscopic surgeries addressing her endometriosis.

**Figure:**
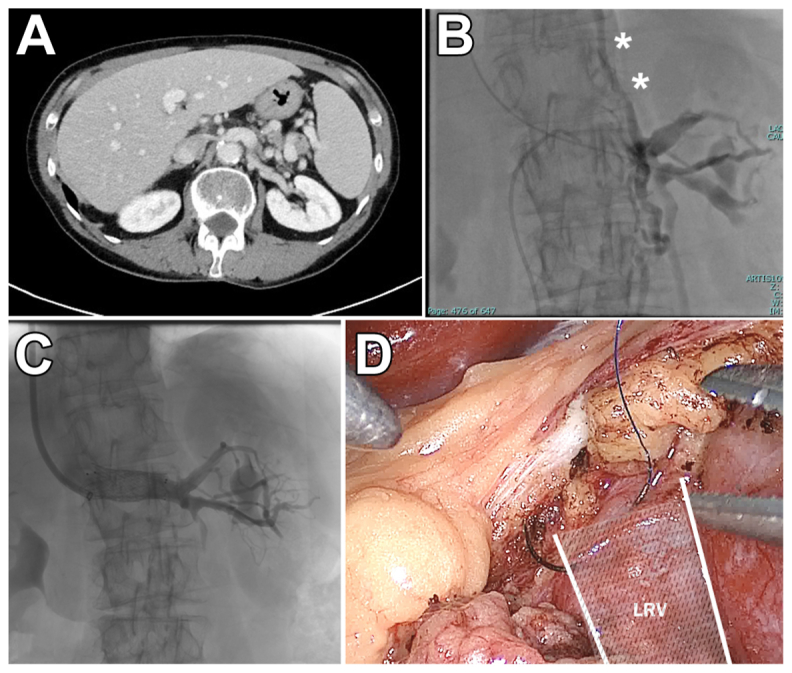

A preoperative computed tomography scan revealed a recurrent Nutcracker syndrome onset (*A*).

Using a dual access approach via the right femoral and left jugular veins, a Venovo stent measuring 12 × 80 mm was successfully placed at the renal level after stenosis confirmation (*B*, *C*). This procedure was conducted under the guidance of fluoroscopy and intravascular ultrasound examination. To finalize the intervention, laparoscopic access was established, incorporating external anchoring points for enhanced stent stability (*D*). IVUS examination was used to confirm the stent positioning. A 6-month follow-up computed tomography scan revealed stent stability, with neither stent fractures/kinks or migration nor symptoms. The patient signed a form consent for intervention and anonymized data dissemination.

Despite renal vein stenting has emerged as a good choice in treating Nutcracker syndrome, owing to its minimally invasive nature, open surgery by transposition may still be considered the best strategy.^1^ However, after open treatment, recurrence requiring reintervention has been described in approximately 30% of cases.^1^ Conversely, the stent's placement at the aortomesenteric level is problematic because the narrow angle and pulsatile nature of the aorta can compromise its stability, causing it to migrate over time.^2^ To mitigate this risk, a novel hybrid technique with laparoscopic stent external fixation has been introduced.^3^ This approach involves securing the stent to the left renal vein with a single transfixing polypropylene stitch, significantly decreasing the likelihood of migration while maintaining low morbidity.

## Funding

None.

## Disclosures

None.

## Figures and Tables

**Cover image:**